# Measurement of fasting breath hydrogen concentration as a simple diagnostic method for pancreatic exocrine insufficiency

**DOI:** 10.1186/s12876-021-01776-8

**Published:** 2021-05-10

**Authors:** Kota Uetsuki, Hiroki Kawashima, Eizaburo Ohno, Takuya Ishikawa, Tadashi Iida, Kenta Yamamoto, Kazuhiro Furukawa, Masanao Nakamura, Takashi Honda, Masatoshi Ishigami, Yoshiki Hirooka, Mitsuhiro Fujishiro

**Affiliations:** 1 Department of Gastroenterology and Hepatology, Nagoya University Graduate School of Medicine, 65 Tsuruma-cho, Showa-ku, Nagoya 466-8550 Japan; 2 Department of Endoscopy, Nagoya University Hospital, 65 Tsuruma-cho, Showa-ku, Nagoya 466-8550 Japan; 3 Department of Gastroenterology and Gastroenterological Oncology, Fujita Health University, 1-98 Dengakugakubo, Kutsukake-cho, Toyoake, Aichi 470-1192 Japan

**Keywords:** Pancreatic exocrine insufficiency, Fasting breath hydrogen concentration, Pancreatic disease, Non-invasive measurement

## Abstract

**Background:**

Pancreatic exocrine insufficiency (PEI) is associated with the outcome of pancreatic disease. However, there is no method for assessing PEI that can be used noninvasively and easily for outpatient. It has been reported that changes in intestinal bacteria caused by PEI may increase breath hydrogen concentration (BHC) levels during glucose or lactose loading. We have evaluated the usefulness of fasting breath hydrogen concentration (FBHC) measurement without glucose loading for the evaluation of PEI.

**Methods:**

Sixty patients underwent FBHC measurement, BT-PABA testing, and microbiome analysis. They were classified into PEI group (PABA excretion rate < 73.4%, n = 30) and non-PEI group (n = 30). The FBHC of the two groups were compared, and the diagnostic ability of PEI by them was evaluated. The 16 s rRNA (V3–V4) from fecal samples was analyzed by MiSeq.

**Results:**

FBHC levels was higher in the PEI group 15.70 (1.4 to 77.0) ppm than in the non-PEI group 2.80 (0.7 to 28.2) ppm (*P* < 0.0001). FBHC was negatively correlated with PABA excretion rate (r =  − 0.523, *P* < 0.001). The cutoff value of FBHC of 10.7 ppm (95% CI: 0.678–0.913, *P* < 0.001) showed a sensitivity of 73.3% and specificity of 83.3% for PEI diagnosis. In the PEI group, there was a significant increase of relative abundance of phylum Firmicutes (*P* < 0.05) and the genus *Clostridium* (*P* < 0.05).

**Conclusion:**

FBHC shows good potential as a simple and repeatable test for the diagnosis of PEI. The elevated FBHC levels may be caused by hydrogen-producing bacteria such as *Clostridium*.

## Background

Pancreatic exocrine insufficiency (PEI) is defined as ‘a state of declined pancreatic enzyme activity in the intestinal lumen to a level below the threshold required to maintain normal digestion’ [1]. In pancreatic disease, PEI occurs at a high frequency and causes indigestion and nutritional deficiency, which have a negative influence on nutritional status and quality of life (QOL) [2]. Pancreatic enzyme replacement therapy (PERT) significantly improves overall survival time, treatment tolerability, symptoms, and QOL of patients with pancreatic diseases such as pancreatic carcinoma (PC) and chronic pancreatitis (CP) [3, 4]. This shows the importance of appropriate diagnosis of PEI and introduction of treatment. There are many diagnostic methods for PEI, but no consensus on the best approach in clinical practice [5]. The BT-PABA test, which is the only method covered by national health insurance in Japan, is a complicated process that requires about a 6-h test time and fasting.

The breath hydrogen test (BHT) for pancreatic disease was first examined in the 1960s, and this test is now mainly utilized as an indirect diagnostic method for small intestinal bacterial overgrowth syndrome (SIBO), which complicates some CP cases [6]. A study in a small number of patients suggested a relationship between PEI and SIBO [7], but the test requires measurement of breath hydrogen continuously for about 2–4 h in a fasting state after glucose loading [8, 9]. Also, patients with a high fasting breath hydrogen concentration (FBHC) were excluded in many studies [10] and there is no consensus on the relationship between FBHC and pancreatic disease [8, 9].

We have found a significant increase in FBHC in patients with pancreatic duct stenosis, which suggests that a decline in exocrine pancreatic secretion induces changes in intestinal bacterial flora [11]. The objective of this study was to clarify the relationships among exocrine pancreatic secretion, FBHC and intestinal bacterial flora, and to investigate the usefulness of measuring FBHC as a simple diagnostic method for PEI.

## Methods

### Subjects

The subjects were patients aged ≥ 20 years old who agreed to participate in the study. The subjects were prospectively collected from April 2019 to June 2020. All subjects were inpatients and were classified into those with PC, CP, other pancreatic diseases, and a normal pancreas. PC cases were histopathologically diagnosed with pancreatic duct cancer by surgery or EUS-guided fine needle aspiration. CP was diagnosed using the M-ANNHEIM criteria [12]. Patients admitted for a disease other than pancreatic disease and in whom pancreatic disease was excluded based on imaging and blood tests were included as subjects with a normal pancreas. The exclusion criteria were pregnancy; patients being fasted long term; use of antibiotics, probiotics, or pancreatic enzyme replacement drugs within one month before the test: history of surgery on the digestive tract or lung; presence of concomitant disease of cancer of other organs, stage 2 or advanced chronic renal failure, decompensated cirrhosis, active pulmonary disease, gastrointestinal obstruction, apparent gastrointestinal hemorrhage, caries being treated, or periodontal disease; no written consent, and judgement as inappropriate by a physician in charge [8, 9].

### Pancreatic function test

The BT-PABA test and the 24-h urinary C peptide excretion (CPR) test were performed as exocrine and endocrine pancreatic secretion tests, respectively. These tests were performed under non-fasting conditions within one week before and after measuring breath hydrogen. In both tests, the measurement was repeated 3 times on different days and the mean was used for analysis [13]. A PABA excretion rate of < 73.4% was regarded as reduced exocrine pancreatic secretion [14], and a CPR rate of < 29.2 µg/day was regarded as reduced endocrine pancreatic secretion.

### Breath sampling

All patients ate a hospital meal on the day before the test and were fasted after 21:00 with drinking of water only. On the day of each breath test, the patients brushed their teeth at 7:00 a.m., breathed deeply twice, and held their breath for 15 s while end-expired breath was collected. Cigarette smoking, alcohol intake, excess exercise, and eating between meals were prohibited after admission. The collection method followed that of the Rome Consensus Conference and North American Consensus in 2017 [8, 9].

### Expired gas analysis

A sensor gas chromatograph (SGHA, Nissha FIS Inc.) was used for measurements. Analysis of the results was performed using specialized SGC Analysis Software. Unlike general GC analysis, quantitation was performed using the peak height (= signal intensity). Hydrogen, carbon monoxide, and methane were measured and the target measurement level was 1.0–100 ppm.

### Sample collection of microbiota and 16S rRNA gene sequencing

Feces collected during hospitalization were rapidly frozen. DNA was isolated from feces using a DNeasy PowerSoil Kit (Qiagen, Hilden, Germany) and amplified by targeting the V3–4 region of bacterial 16S rRNA using universal primers (forward: 5′-TCG TCG GCA GCG TCA GAT GTG TAT AAG AGA CAG CCT ACG GGN GGC WGC AG-3′ and reverse: 5′-GTC TCG TGG GCT CGG AGA TGT GTA TAA GAG ACA GGA CTA CHV GGG TAT CTA ATC C-3′). The PCR products were pooled, and sequencing libraries were constructed and sequenced using an Illumina MiSeq sequencer. Pair-End Reads were prepared using MiSeq Reagent Kit v3 with 2 × 300 reads and 600 cycles (Illumina, San Diego, CA, USA). Analysis of 16S rRNA gene sequence data was performed using USEARCH 6.1, Microbial Ecology (QIIME 1.9.1) and Greengenes v.13_8.

### Analytical methods

For between-group comparison based on the BT-PABA test, the subjects were classified into PEI and non-PEI groups based on the criterion of a PABA excretion rate of 73.4%. Associations of the PABA excretion rate were examined with age, height, body mass index (BMI), pancreatic disease (PC, CP, and other pancreatic diseases), rates of concomitant diseases (hypertension, dyslipidemia, diabetes), history of alcohol intake, blood test findings (hemoglobin, HbA1c, creatinine, urea nitrogen, amylase, lipase, total protein, albumin, CEA, CA19-9), and presence of characteristic imaging findings in pancreatic disease (pancreatic hypertrophy, calcification, pancreatic cyst, main pancreatic duct (MPD) stenosis, and MPD dilatation). Concomitant diseases were defined as follows: hypertension, ≥ 140/90 mmHg blood pressure or treatment with an oral hypotensive drug; dyslipidemia, ≥ 160 mg/dL LDL cholesterol or treatment with an LDL-lowering drug; and diabetes, ≥ 6.5% or higher hemoglobin A1c or under treatment [15, 16]. Regarding alcohol ingestion, a subject with a history of continuous ingestion of ≥ 80 g pure ethanol a day [17] was defined as a heavy drinker. Pancreatic hypertrophy was defined using the criteria of Haage et al., in which the thicknesses of the pancreatic head and tail correspond to one or more vertebral bodies and 2/3 or more of the vertebral body, respectively [18]. A MPD with a diameter > 3 mm was regarded as MPD dilatation. MPD stenosis was diagnosed using endoscopic retrograde cholangiopancreatography (ERCP), magnetic resonance cholangio pancreatgraphy (MRCP), and endoscopic ultrasound (EUS), and calcification and a pancreatic cyst were diagnosed using computed tomography (CT) and EUS.

The relationship between endocrine pancreatic secretion and fasting expired gas levels was investigated based on the associations of FBHC, fasting breath carbon monoxide concentration (FBCC), and fasting breath methane concentration (FBMC) with pancreatic function. For exocrine pancreatic secretion, intestinal bacterial flora were compared between the PEI and non-PEI groups.

### Statistical analysis

Statistical analysis was performed using SPSS v.27.0 (IBM Corp.). All tests were 2-sided and *P* < 0.05 was regarded as significant. Continuous variables were analyzed as the median and range. Comparison of data that were not normally distributed was with a non-parametric Mann–Whitney-U test. Differences in rates between two groups were examined by Fisher exact test. Correlations between expired gas levels and pancreatic function tests were analyzed using a Spearman correlation coefficient (r). The cut-off value for FBHC for diagnosis of PEI was determined from a receiver operating characteristic (ROC) curve and the area under this curve (AUCROC), so as to maximize the Youden index (sensitivity + specificity-1). For between-group comparison of intestinal bacteria, LEfSe (http://huttenhower.sph.harvard.edu/galaxy/) was used with default settings.

## Results

### Patient background and clinical parameters

Of 80 patients who were enrolled in the study between April 2019 and June 2020, the items described above could be analyzed in 60. The excluded patients were those for whom feces could not be acquired during hospitalization (n = 4), intestinal bacterial flora could not be analyzed due to the poor condition of fecal samples (n = 8), and antibiotic treatment was initiated before expired gas collection after admission (n = 8). The 60 patients were divided into PEI and non-PEI groups (n = 30 each) based on a PABA excretion rate of 73.4% (Table 1). The PEI group had a significantly higher rate of heavy drinkers (*P* = 0.023), a significantly lower level of serum albumin (*P* = 0.006), and a significantly higher rate of main pancreatic duct stenosis on imaging (*P* = 0.038). In contrast, the image findings of pancreas were more frequently normal in patients in the non-PEI group (*P* = 0.020).

**Table 1:** Patient background and clinical parameters

	PEI group (n = 30)	Non-PEI group (n = 30)	*P* value
Age, median [range]	71.0 [46–87]	71.0 [40–81]	1.000
Sex, male: female	24: 6	22: 8	0.542
BMI, median [range]	21.585 [15.99–28.07]	21.090 [15.24–26.38]	0.255
History of alcohol intake, n (%)	19 (63.3)	19 (63.3)	1.000
Heavy drinkers, n (%)	7 (23.3)	1 (3.3)	0.023
Smoking history, n (%)	17 (56.7)	19 (63.3)	0.598
Pancreatic disease			
PC, n (%)	11 (36.7)	12 (40.0)	0.791
CP, n (%)	8 (26.7)	3 (10.0)	0.095
Other pancreatic disease, n (%)	11 (36.7)	10 (33.3)	0.761
Concomitant diseases			
Hypertension, n (%)	10 (33.3)	15 (50.0)	0.190
Dyslipidemia, n (%)	10 (33.3)	11 (36.7)	0.787
Diabetes, n (%)	14 (46.7)	13 (43.3)	0.795
Blood test findings			
Hb (g/dL) [range]	13.50 [7.9–15.5]	13.50 [10.9–15.9]	0.877
BUN (mg/dL) [range]	16.05 [3.7–29.2]	15.75 [6.2–26.9]	1.000
Cre (mg/dL) [range]	0.775 [0.49–1.32]	0.730 [0.42–1.24]	0.225
TP (g/dL) [range]	7.00 [5.5–8.0]	7.20 [6.2–7.8]	0.716
Alb (g/dL) [range]	3.95 [1.4–4.5]	4.20 [3.6–4.7]	0.006
Amy (IU/L) [range]	83.5 [30–374]	74.5 [36–350]	0.877
Lipase (IU/L) [range]	48.5 [4–781]	34.5 [10–889]	0.706
HbA1c (NGSP %) [range]	6.65 [4.9–13.7]	6.20 [4.9–10.5]	0.195
CEA (ng/mL) [range]	3.2 [1.0–44.4]	2.6 [0.7–105.8]	0.261
CA19-9 (U/mL) [range]	119.50 [< 1–23210]	61.50 [< 1–197000]	0.446
Imaging findings			
Pancreatic hypertrophy, n (%)	9 (30.0)	3 (10.0)	0.053
Calcification, n (%)	4 (13.3)	2 (6.7)	0.389
Pancreatic cyst, n (%)	6 (20.0)	5 (16.7)	0.739
MPD dilatation, n (%)	19 (63.3)	13 (43.3)	0.121
MPD stenosis, n (%)	20 (66.7)	12 (40.0)	0.038
Normal pancreas, n (%)	0 (0.0)	5 (16.7)	0.020

*PEI* pancreatic exocrine insufficiency, *BMI* body mass index, *PC* pancreatic carcinoma, *CP* chronic pancreatitis, *Hb* hemoglobin, *BUN* blood urea nitrogen, *Cre* creatinine, *TP* total protein, *Alb* albumin, *Amy* amylase, *HbA1c* hemoglobin A1c, *NGSP* national glycohemoglobin standardization program, *CEA* carcinoembryonic antigen, *CA19-9* carbohydrate antigen 19-9, *MPD* major pancreatic duct

### Expired gas analysis

**Fig. 1:**
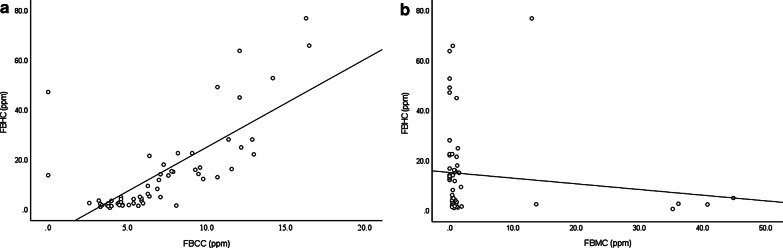
Correlation of fasting breath hydrogen concentration (FBHC) with fasting breath carbon monoxide concentration (FBCC) and fasting breath methane concentration (FBMC). **a** Fasting breath hydrogen concentration (FBHC) and fasting breath carbon monoxide concentration (FBCC). There was a strong positive correlation between FBHC and FBCC (r = 0.754, *P* < 0.001). **b** Fasting breath hydrogen concentration (FBHC) and fasting breath methane concentration (FBMC). There was a negative correlation between FBHC and FBMC (r =  − 0.387, *P* = 0.001)

**Fig. 2:**
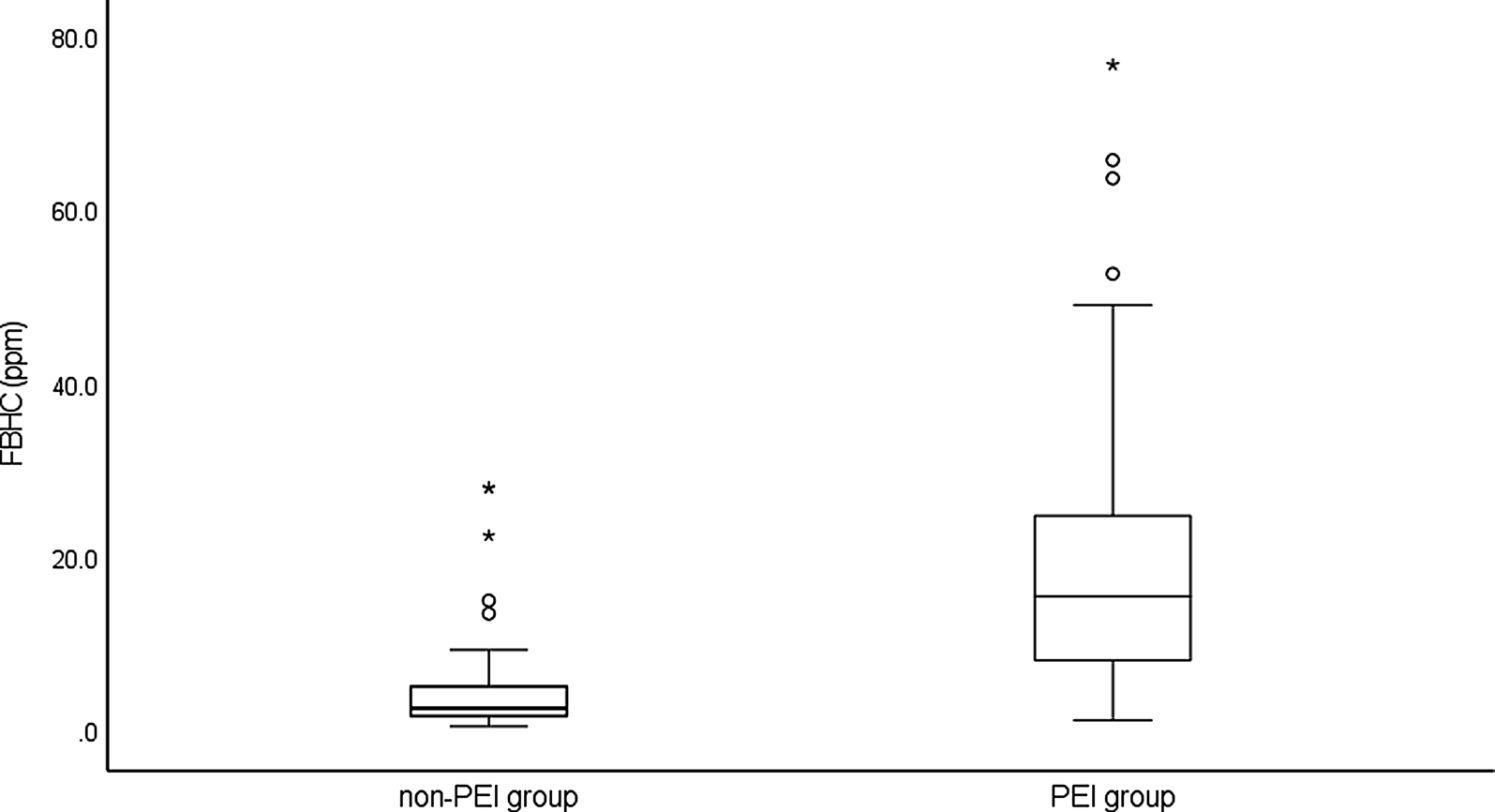
Box plot of fasting breath hydrogen concentration (FBHC) in the exocrine pancreatic secretion test. Box plot of fasting breath hydrogen concentration (FBHC) in non-pancreatic exocrine insufficiency (non-PEI) and PEI groups. The median FBHC of non-PEI and PEI groups were 2.80 ppm and 15.70 ppm, respectively (*P* < 0.001)

**Fig. 3:**
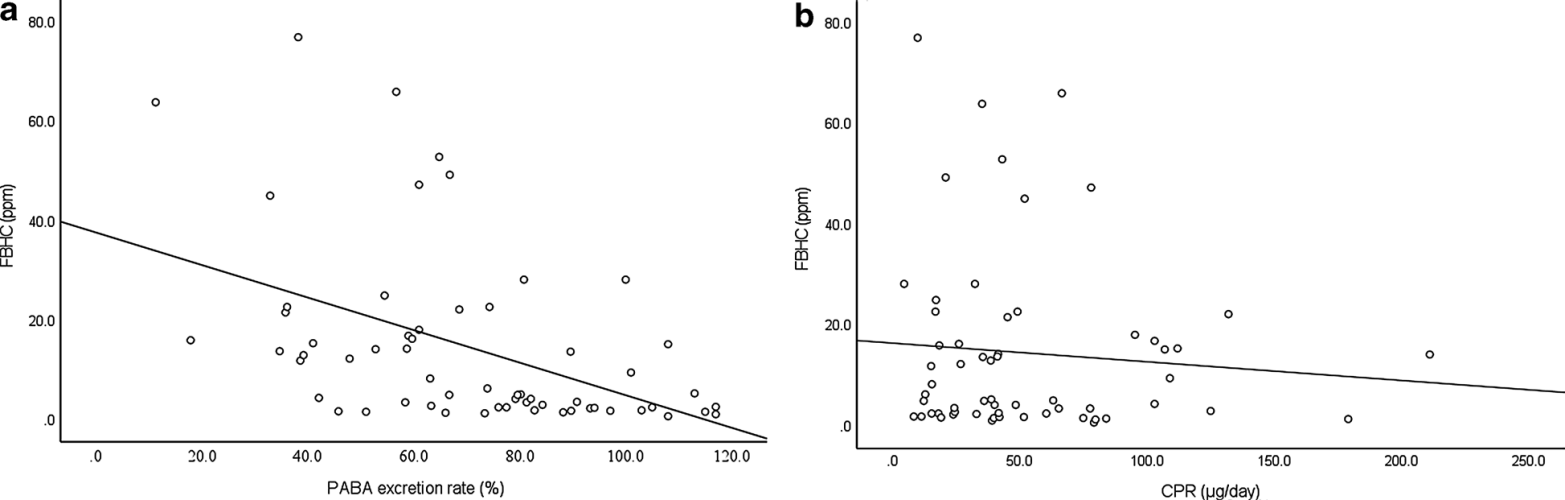
Correlation between breath hydrogen concentration (FBHC) and pancreatic function. **a** The result of BT-PABA test. FBHC had a negative correlation with PABA excretion rate (r =  − 0.523, *P* < 0.001). **b** The result of 24 urinary C peptide excretion (CPR) test. There was no significant correlation between FBHC and CPR (r =  − 0.067, *P* = 0.608)

**Fig. 4:**
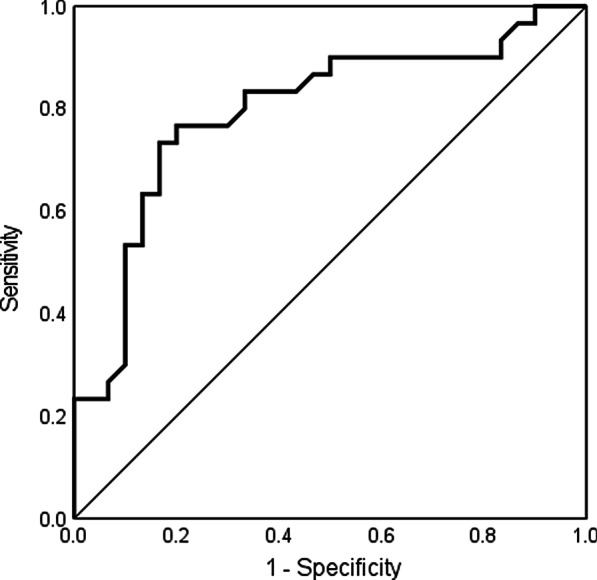
Diagnostic accuracy of pancreatic exocrine insufficiency (PEI) due to elevated fasting breath hydrogen concentration (FBHC) levels. An receiver operator characteristic (ROC) curve for fasting breath hydrogen concentration (FBHC) and PABA excretion rate. The cut-off value for the highest sensitivity with maintaining specificity was 10.7 ppm for diagnosis of PEI (the area under ROC (AUCROC): 0.796, 95% confidence interval: 0.678–0.913, *P* < 0.001)

There was a strong positive correlation between FBHC and FBCC (r = 0.754, *P* < 0.001) and a negative correlation between FBHC and FBMC (*r* =  − 0.387, *P* = 0.001) (Fig. 1a, b). FBHC was significantly higher in the PEI group than in the non-PEI group (median: 2.80 (0.7–28.2) vs. 15.70 (1.4–77.0) ppm, *P* < 0.001) (Fig. 2). Similarly, FBCC was significantly higher in the PEI group (*P* = 0.001), whereas there was no significant difference in FBMC (*P* = 0.216) (Table 2). FBHC had a negative correlation with PABA excretion rate (*r* =  − 0.523, *P* < 0.001) and no correlation with the CPR value (*r* =  − 0.067, *P* = 0.608) (Fig. 3a, b). FBCC showed similar respective correlations (r =  − 0.462, *P* < 0.001; r =  − 0.091 *P* = 0.487) since it was positively correlated with FBHC. FBMC was not correlated with the PABA excretion rate (*r* = 0.131, *P* = 0.320) or CPR value (*r* = 0.081, *P* = 0.541). An ROC curve for FBHC and PABA excretion rate was used to determine a cut-off value for FBHC (Fig. 4). A cut-off of 10.7 ppm (AUCROC: 0.796, 95% confidence interval: 0.678–0.913, *P* < 0.001) gave a sensitivity of 73.3% and a specificity of 83.3% for diagnosis of PEI.

**Table 2:** Comparison of expired gas in PEI and non-PEI groups

	PEI group (n = 30)	Non–PEI group (n = 30)	*P* value
FBHC (ppm) [range]	15.70 [1.4–77.0]	2.80 [0.7–28.2]	< 0.001
FBCC (ppm) [range]	8.15 [0.0–16.5]	4.90 [2.6–12.9]	0.001
FBMC (ppm) [range]	0.55 [0.0–36.2]	0.70 [0.0–44.9]	0.216

*PEI* pancreatic exocrine insufficiency, *FBHC* fasting breath hydrogen concentration, *FBCC* fasting breath carbon monoxide concentration, *FBMC* fasting breath methane concentration, *ppm* parts per million

### PABA excretion rate-associated changes in intestinal bacterial flora

**Fig. 5:**
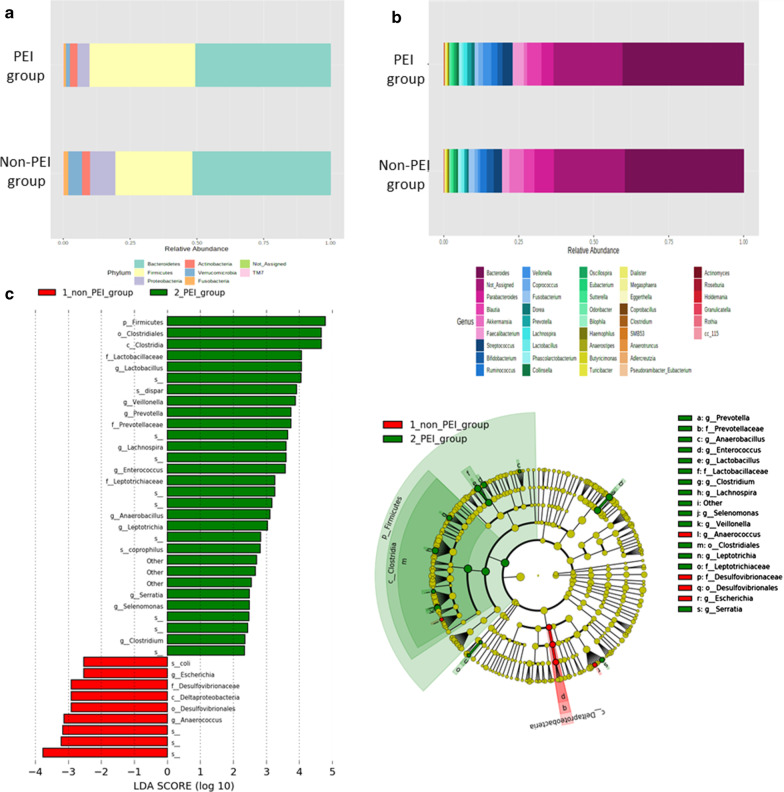
The composition and comparison of bacteria in non-PEI and PEI groups. Stacked bar chart comparing microbiome at the phylum level (**a**) and genus level (**b**) in the non-PEI and PEI groups. **c** Comparison of microbiomes between groups. Bacterial names shown with green bars had significantly higher relative abundance in the PEI group than in the non-PEI group, and bacterial names shown with red bars had significantly lower relative abundance in the PEI group than in the non-PEI group. A comparison of microbiomes showed increases in the genera *Clostridium*, *Lachnospira*, *Veillonella*, *Selenomonas*, and Anaerococcus in the PEI group (*P* < 0.05). Increases in the genera *Enterococcus* and *Lactobacillus*, and genera *Leptotrichia*, *Prevotella*, *Serratia*, and *Aggregatibacter* were also noted (*P* < 0.05)

The bacteria proportions at the ‘phylum’ level in the PEI and non-PEI groups are shown in Fig. 5a. Bacteroidetes was dominant in both groups. The occupancy by the phylum Firmicutes was higher in the PEI group, and those for Proteobacteria, Verrucomicrobia, and Fusobacteria were lower. At the ‘genus’ level, the occupancies by the genera *Blautia*, *Faecalibacterium*, and *Streptococcus* were higher and those of *Parabacteroides* and *Akkermansia* were low in the PEI group (Fig. 5b). A comparison of microbiomes showed increases in the genera *Clostridium*, *Lachnospira*, *Veillonella*, *Selenomonas*, and *Anaerococcus* belonging to the class Clostridia, which are obligate anaerobes, in the PEI group (*P* < 0.05). Increases in the genera *Enterococcus* and *Lactobacillus* belonging to the family *Lactobacillaceae*, and genera *Leptotrichia*, *Prevotella*, *Serratia*, and *Aggregatibacter* belonging to the family *Leptotrichiaceae* were also noted (Fig. 5c) (*P* < 0.05).

## Discussion

PERT can improve the quality of life and life expectancy of PEI patients [3, 4]. However, not all patients with pancreatic diseases are being evaluated for PEI because complicated tests are required for its diagnosis. PERT is also difficult for all patients because it needs much cost and many tablets. In order to provide PERT to appropriate patients, the development of a diagnosis of PEI that is simple and can be easily performed in an outpatient is required.

BHT has the potential to diagnose PEI. In humans, hydrogen is only produced in the intestine through degradation and metabolism of unabsorbed food by intestinal bacteria, and about 14% is excreted via expiration [19]. Since a decline in exocrine pancreatic secretion is known to induce changes in intestinal bacterial flora, this study was performed with the hypothesis that simple FBHC measurement may be useful to predict PEI.

In this study, FBHC was significantly higher in the PEI group and had a negative correlation with the PABA excretion rate. Thus, the breath hydrogen concentration increased as exocrine pancreatic secretion decreased. The normal limit of FBHC is unclear, but this value varies from 10 to 20 ppm [20]. At present, ≥ 20 ppm, the diagnostic criterion for SIBO, is the accepted value [8, 9, 20], but FBHC ≥ 20 ppm was found in only 12 of 30 patients in the PEI group (and in none in the non-PEI group). This suggests that ≥ 20 ppm is inappropriate as a screening criterion because it may increase the false negative rate. A cut-off value of FBHC of 10.7 ppm gave favorable sensitivity and specificity for diagnosis of PEI in this study.

Regarding other expired gas, cross-sensitivity of FBCC with the hydrogen concentration has been shown [21] and a strong positive correlation with FBHC was found in the current study. FBHC has been found to decrease in subjects with high FBMC, and these variables had an inverse correlation in this study. Fewer of our subjects produced methane compared with the rate of 44% in a previous study, and we did not find an association of FBMC with pancreatic function [22].

To elucidate this mechanism, we also focused on the relationship between FBHC and microbiome. In comparison of intestinal bacterial flora, as previously reported, increases in the division Firmicutes [23, 24], genus *Clostridium* [25], and family *Lactobacillaceae* [26] were observed in PC, CP, and PEI. Of 343 species of intestinal bacteria registered in the Human Microbiome Project, about 71% encode hydrogenase, an enzyme catalyzing a reversible oxidation–reduction reaction of hydrogen. The division Firmicutes, which significantly increased in the PEI group, accounts for 21% of the hydrogenase content in bacterial flora in the human colon [27]. The genus *Clostridium* is a representative hydrogen-producing bacteria producing 1.1–2.3 mol H_2_/mol glucose at temperatures (30–40 °C) close to that of the human body and has been widely studied in the biomass field [28, 29].

The genus *Clostridium* is one of the main hydrogen sources in the human intestine [30] and is likely to have been the cause of elevation of FBHC in the PEI group. In addition, some of Clostridium spp. form a carbohydrate-associated enzyme complex (a “cellulosome”) and 57 cellulosome genes encoding lipase, peptidase, and proteinase inhibitors, in addition to carbohydrate-activating enzymes, have been discovered [31]. Since undigested food flows in the intestine due to PEI, Clostridium with these characteristics may have increased and resulted in an increase in hydrogen. The genus *Lactobacillus* of the family *Lactobacillaceae* also produces hydrogen in a medium temperature environment [32], and a state with reduced exocrine secretion (i.e., an intestinal condition with undigested food) may be an advantageous environment for a hydrogen-producing anaerobe.

PERT has been shown to improve undernutrition, gastrointestinal symptoms, and QOL by decreasing undigested food [4]. This suggests that an increase in undigested food is reflected as FBHC elevation, suggesting its potential as an effective pre-test marker before use of PERT. A bag is the only running cost required for breath hydrogen measurement, which reduces the cost. The test time is about 1 min and the measurement time is about 4 min, which are shorter than those in other tests, and the test is non-invasive and can be performed repeatedly. Thus, it may also be useful for judgment of the effect of PERT, as well as for screening prior to treatment.

Several diagnoses of PEI have been used clinically. Faecal elastase-1 (FE-1) has been used as a reliable diagnostic method for PEI, but a faecal sample is required [33]. Faecal collection can be difficult for outpatients, especially for the elderly patients [34]. In addition, the sensitivity of FE-1 has been reported to be low in mild to moderate PEI patients [33]. The 13C-Mixed Triglyceride Breath Test (13C-MTBT) has been reported to be potentially useful [35]. However, there is not enough evidence for clinical application, and it requires long examination time and fasting. We compared FBHC with BT-PABA as a control, but could not compare it with other tests because of the limitation of public insurance in Japan. However, we believe that FBHC is cheaper and easier than these methods, and is useful for early diagnosis of PEI.

There are several limitations in this study. First, all subjects were inpatients with diseases, and no samples were obtained from healthy persons. Therefore, we could not eliminate the confounding factors such as alcohol consumption, smoking, and diabetes. that may have affected the results of the BHT and microbiome analysis [36, 37]. Second, there were only a few patients with FBMC elevation and the clinical significance of this effect could not be analyzed. Production of methane consumes hydrogen and this may latently influence hydrogen measurement [22]. Third, we could not clarify the relationship with the concept of SIBO because we did not use glucose or lactose loading for BHT. We used the average value of three times to increase the reliability of the score, but the accuracy of FBHC for PEI diagnosis did not meet our expectations at the present setting. It is necessary to increase the number of patients including healthy controls and measure both fasting and glucose loaded hydrogen breath levels to solve these problems.

## Conclusion

Patients with PEI have elevated FBHC levels, which may be related to the genus Clostridium producing hydrogen in the gut. FBHC measurement may be useful in the diagnosis of PEI.

## Data Availability

The data of this study are available from the corresponding author upon reasonable request.

## Abbreviations

PEI: Pancreatic exocrine insufficiency
FBHC: Fasting breath hydrogen concentration
CI: Confidence interval
QOL: Quality of life
PERT: Pancreatic enzyme replacement therapy
PC: Pancreatic carcinoma
CP: Chronic pancreatitis
BHT: Breath hydrogen test
SIBO: Small intestinal bacterial overgrowth syndrome
CPR: The 24-h urinary C peptide excretion test
BMI: Body mass index
MPD: Main pancreatic duct
ERCP: Endoscopic retrograde cholangiopancreatography
MRCP: Magnetic resonance cholangiopancreatgraphy
EUS: Endoscopic ultrasound
CT: Computed tomography
FBCC: Fasting breath carbon monoxide concentration
FBMC: Fasting breath methane concentration
ROC: Receiver operating characteristic
